# Supplementary material for: Venomix: a simple bioinformatic pipeline for identifying and characterizing toxin gene candidates from transcriptomic data
Source: PeerJ. 2018 Jul 31;6:e5361. doi: 10.7717/peerj.5361 (PMC6074769; doi:10.7717/peerj.5361)
Supplement: Supplemental Information 3 [file peerj-06-5361-s003.gz › FinalOutput_E-6/Cysteine-rich_venom_protein_25-A_6/finaltree.pdf]

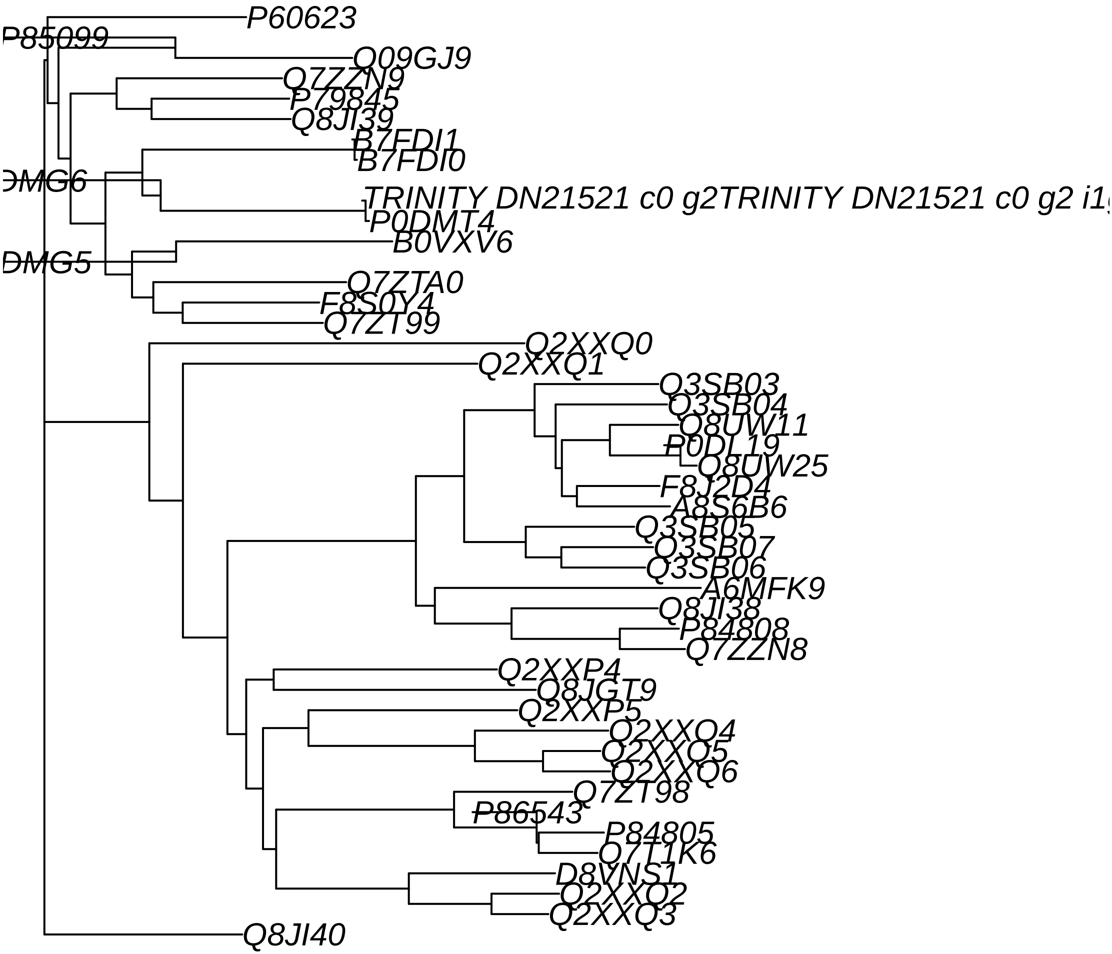
P60623
P85099
Q09GJ9
Q7ZZN9
P79845
Q8JI39
B7FDI1
B7FDI0
DMG6
TRINITY DN21521 c0 g2TRINITY DN21521 c0 g2 i1
P0DMT4
B0VXV6
DMG5
Q7ZTA0
F8S0Y4
Q7ZT99
Q2XXQ0
Q2XXQ1
Q3SB03
Q3SB04
Q8UW11
P0DL19
Q8UW25
F8J2D4
A8S6B6
Q3SB05
Q3SB07
Q3SB06
A6MFK9
Q8JI38
P84808
Q7ZZN8
Q2XXP4
Q8JGT9
Q2XXP5
Q2XXQ4
Q2XXQ5
Q2XXQ6
Q7ZT98
P86543
P84805
Q7T1K6
D8VNS1
Q2XXQ2
Q2XXQ3
Q8JI40
